# Measurable Metrics of Mesenchymal Stem Cell Aging

**DOI:** 10.17691/stm2025.17.5.01

**Published:** 2025-10-31

**Authors:** D.A. Kalashnikova, S.E. Romanov, D.A. Maksimov, I.A. Plokhikh, R.Yu. Epifanov, R.I. Mullyadjanov, L.O. Sidelnikov, P.A. Antoshina, Ya.A. Osipov, V.V. Shloma, A.A. Budilina, E.M. Samoylova, V.P. Baklaushev, P.P. Laktionov

**Affiliations:** Junior Researcher, Laboratory of Epigenetics; Novosibirsk National Research State University, 1 Pirogova St., Novosibirsk, 630090, Russia; Junior Researcher, Laboratory of Genomics; Institute of Molecular and Cellular Biology of the Siberian Branch of the Russian Academy of Sciences, 8/2 Acad. Lavrentiev Avenue, Novosibirsk, 630090, Russia; PhD, Researcher, Laboratory of Epigenetics; Novosibirsk National Research State University, 1 Pirogova St., Novosibirsk, 630090, Russia; Researcher, Laboratory of Genomics; Institute of Molecular and Cellular Biology of the Siberian Branch of the Russian Academy of Sciences, 8/2 Acad. Lavrentiev Avenue, Novosibirsk, 630090, Russia; PhD, Senior Researcher, Laboratory of Epigenetics; Novosibirsk National Research State University, 1 Pirogova St., Novosibirsk, 630090, Russia; Researcher, Laboratory of Genomics; Institute of Molecular and Cellular Biology of the Siberian Branch of the Russian Academy of Sciences, 8/2 Acad. Lavrentiev Avenue, Novosibirsk, 630090, Russia; Junior Researcher, Laboratory of Applied Digital Technologies; Novosibirsk National Research State University, 1 Pirogova St., Novosibirsk, 630090, Russia; Junior Researcher, Laboratory of Applied Digital Technologies; Novosibirsk National Research State University, 1 Pirogova St., Novosibirsk, 630090, Russia; DSc, Head of the Laboratory of Applied Digital Technologies; Novosibirsk National Research State University, 1 Pirogova St., Novosibirsk, 630090, Russia; Head of the Laboratory of Supercomputing and Artificial Intelligence in Power Engineering; Kutateladze Institute of Thermophysics of the Siberian Branch of the Russian Academy of Sciences, 1 Acad. Lavrentiev Avenue, Novosibirsk, 630090, Russia; Research Assistant, Laboratory of Epigenetics; Novosibirsk National Research State University, 1 Pirogova St., Novosibirsk, 630090, Russia; Junior Researcher, Laboratory of Genomics; Institute of Molecular and Cellular Biology of the Siberian Branch of the Russian Academy of Sciences, 8/2 Acad. Lavrentiev Avenue, Novosibirsk, 630090, Russia; Research Assistant, Laboratory of Epigenetics; Novosibirsk National Research State University, 1 Pirogova St., Novosibirsk, 630090, Russia; PhD, Head of the Laboratory of Genomics; Institute of Molecular and Cellular Biology of the Siberian Branch of the Russian Academy of Sciences, 8/2 Acad. Lavrentiev Avenue, Novosibirsk, 630090, Russia; Research Assistant, Laboratory of Molecular Mechanisms of Regeneration and Aging; Engelgard Institute of Molecular Biology of the Russian Academy of Sciences, 32 Vavilova St., Moscow, 119991, Russia; Junior Researcher, Laboratory of Regenerative Medicine; Research Institute of Pulmonology of Federal Medico-Biological Agency of Russia, 28, Bldg. 10, Orekhoviy Blvd., Moscow, 115682, Russia; Junior Researcher, Laboratory of Epigenetics; Novosibirsk National Research State University, 1 Pirogova St., Novosibirsk, 630090, Russia; Junior Researcher, Laboratory of Molecular Mechanisms of Regeneration and Aging; Engelgard Institute of Molecular Biology of the Russian Academy of Sciences, 32 Vavilova St., Moscow, 119991, Russia; MD, DSc, Head of the Laboratory of Molecular Mechanisms of Regeneration and Aging; Engelgard Institute of Molecular Biology of the Russian Academy of Sciences, 32 Vavilova St., Moscow, 119991, Russia; Head of the Laboratory of Regenerative Medicine; Research Institute of Pulmonology of Federal Medico-Biological Agency of Russia, 28, Bldg. 10, Orekhoviy Blvd., Moscow, 115682, Russia; Chief of the Center for Biomedical Research; Federal Scientific Clinical Center for Specialized Types of Medical Care and Medical Technologies of the Federal Medico-Biological Agency of Russia, 28, Bldg. 1–9, Orekhoviy Blvd., Moscow, 115682, Russia; Head of the Department of Cellular Preparation Development; Federal Center of Brain Research and Neurotechnologies of the Federal Medico-Biological Agency of Russia, 1, Bldg. 10, Ostrovityanova St., Moscow, 117513, Russia; PhD, Senior Researcher, Laboratory of Epigenetics; Novosibirsk National Research State University, 1 Pirogova St., Novosibirsk, 630090, Russia; Senior Researcher, Laboratory of Genomics; Institute of Molecular and Cellular Biology of the Siberian Branch of the Russian Academy of Sciences, 8/2 Acad. Lavrentiev Avenue, Novosibirsk, 630090, Russia

**Keywords:** mesenchymal stem cells, cell senescence, telomeres, expression, predictive models for senescence assessments

## Abstract

**The aim of the study is** to analyze the manifestation of selected cellular senescence markers on the models of replicative senescence, stress-induced senescence, and chronological aging of human mesenchymal stem cells and to study the feasibility of predictive models for assessing the age and duration *in vitro* cultivation based on the transcriptomic data and investigation of cell morphology.

**Materials and Methods:**

In the study, the dynamics of expression of individual genes encoding key regulators of cellular aging across various models of cellular senescence, as well as telomere length were investigated by real-time PCR. The analysis of the high- throughput transcriptome sequencing datasets of mesenchymal stem cells from the donors of different ages has been performed. Using regression methods, predictive models based on transcriptomic data were developed to estimate chronological age and the duration of *in vitro* cultivation. Using microscopy methods and subsequent image analysis by machine-learning algorithms, morphological alterations associated with cellular senescence have been explored and segmentation neural network model has been created for extracting nuclear morphology parameters and classification of the cells based on the duration of cultivation *in vitro*.

**Results:**

*CDKN1A*, *LMNB1*, *HMGB2* genes demonstrated reproducible similar dynamics on the models of replicative or stress-induced senescence and chronological aging of mesenchymal stem cells. The expression profile of the senescence-associated inflammatory phenotype components was variable in different models of cell aging. The analysis of mesenchymal stem cell transcriptomes from the donors of various ages revealed considerable donor-dependent heterogeneity of the cells, which complicates the development of precise transcriptome data-based predictive models. Investigation of the changes in the telomere length has demonstrated its applicability for assessing the dynamics of replicative senescence *in vitro*. The developed segmentation neural network model allowed for detecting senescence-associated dynamics of nuclear morphology alterations in the process of replicative aging.

## Introduction

Preparations of allogenic and autologous mesenchymal stem cells (MSCs) are considered as a promising component of regenerative cell therapy [1]. In clinical trials, high doses reaching hundreds of millions of cells per one procedure are used. It often requires the expansion of the culture *in vitro*, which, together with the compromised functional state of the donor’s organism, may reduce the quality of cell preparations [2–4]. Functional methods of MSC preparation for clinical application imply the assessment of morphology, cell survival, differentiation potential, and biological safety [5, 6]. At the same time, additional investigations are needed to study the effect of functional state of the donor’s organism, cultivation conditions, and duration on the quality of cellular products. When classifying the markers of aging, various functional manifestations such as metabolic and immune disorders, genome instability, and epigenome alterations are distinguished [7]. One of the hallmarks of aging is the accumulation of the senescent cells unable to proliferate, resistant to apoptosis, and possessing the characteristic morphological and metabolic phenotype [3, 7].

Inducers of cellular senescence may include the exhaustion of proliferative potential, accompanied by critical telomere shortening; exposure to toxic, genotoxic, and oxidative stress; induction of oncogenes; inflammation; mitochondrial dysfunction; disruption of epigenetic regulatory mechanisms, and other factors [3, 8–11]. It is important to note that the listed factors may also be the secondary effects of cellular aging, and its phenotypic manifestations at the cellular level can vary in a wide range.

Due to its heterogeneity both *in vitro* and *in vivo*, there is no specific universal marker of cell senescence [12, 13]. Therefore, the investigation of cellular aging dynamics in general relies on the analysis of several markers, whose combination is inherent to this process [3, 12]. These markers include the induction of senescence-associated β-galactosidase, activation of the cell cycle inhibitors p16^INK4A^, p21^CIP1^; reduced expression of LMNB1 and HMGB2 proteins, which shape the structure and architecture of the cell nucleus [14–16]. In addition to the analysis of the mentioned markers, functional tests can be performed to assess proliferative potential, presence of the DNA damage or apoptosis markers [12, 17].

The characteristic feature of the senescent cells is secretion of proinflammatory cytokines, chemokines, growth factors, and proteases, which compose specific senescence-associated secretory phenotype (SASP) [18]. The detection of SASP factors serve as an indicator of cell aging, however, their abundance varies significantly and depends, in particular, on functional cause of the cell senescence [11]. Thus, a classic approach to the exploration of cell senescence is based on the analysis of sufficiently wide spectrum of non-exclusive (non-specific) markers and conducting functional tests. At the same time, the perspective integral assessment of cell senescence by predictive models built on the analysis of DNA methylation patterns, transcriptomic data, and cell morphology is presently being developed [19–21]. These predictive models may consider variability of cell aging phenotype and usually depend, to the lesser extent, on separate markers, which makes them a promising analytical tool.

**The aim of the study** is to analyze manifestations of selected markers of senescence on the models of replicative senescence, stress-induced senescence, and chronological aging of human mesenchymal stem cells. Among the markers we assessed were the level of expression of individual genes whose expression dynamics are associated with aging, global transcriptome alteration during chronological and *in vitro* cellular aging; telomere length measurement and changes in cell morphology and manifestations of specific cytological markers of cellular aging. In this study, we evaluated the applicability of these markers for assessment of MSC aging as well as the limitations of methods used that could potentially bias the analysis. In addition, we have analyzed the conceptual feasibility of building predictive models for estimating chronological age and duration of *in vitro* cultivation based on the transcriptomic data and cell morphology analysis.

## Materials and Methods

### Cell cultures

Cell samples were obtained from the donors with prior written informed consent. The study was approved by the local ethics committee of the Federal Center of Brain Research and Neurotechnologies of the Federal Medico-Biological Agency of Russia (Protocol No.7-5-22 of September 6, 2022).

In the study, MSCs (n=2) isolated from the Wharton’s jelly of the healthy pregnant woman (38–40 weeks of gestation); bone marrow derived MSCs (BM-MSCs) from healthy donors aged 18–25 years (n=3) and donors older than 65 years (n=3) obtained from the mononuclear cell fraction of bone marrow, which was isolated by gradient centrifugation (20 min, 400 g) in the ficoll solution (PanEco, Russia). The cells were cultivated in the DMEM/F12 medium (Servicebio, China) supplemented with 15% fetal bovine serum (Capricorn, Germany) and the antibiotic cocktail of penicillin (100 units/ml) and streptomycin (100 μg/ml) (Gibco, USA). Subculturing was performed at split ratio of 1:4. Using flow cytometry, the expression of the following MSC markers was analyzed: CD29, CD44, CD73, CD90, CD105, CD34, CD45 (FITC/PE; Miltenyi Biotec, Germany), and HLA-DR. The cells exhibited morphology and immunophenotype characteristic of MSCs: CD29^+^, CD44^+^, CD73^+^, CD90^+^, CD105^+^, CD34^–^, CD45^–^.

For induction of the stress-induced cellular senescence, MSCs were cultured until they reached 60% of confluence, after which the culture medium was replaced with medium containing 200 μM hydrogen peroxide (Dia-m, Russia). After 4 h of incubation, the medium was removed, and MSCs were washed twice with the phosphate-saline buffer. Then the cells were incubated under the standard cultivation conditions for 3 days, after which they were used for further analysis.

### Immunostaining and cytochemical analysis of senescence-associated β-galactosidase

Cells were cultured in 96-well plates for confocal microscopy (SPL Lifesciences, South Korea) or on cover glasses precoated with 0.1% gelatin solution (Sigma-Aldrich, USA). Upon reaching the required confluency, the samples were fixed in 4% formaldehyde solution (Sigma-Aldrich, USA). The activity of senescence- associated β-galactosidase was analyzed using the previously described method [22]. For immunostaining, the cells were incubated in the 0.1% Triton X-100 solution (Amresco, USA) for 30 min, after which they were incubated in 1% BSA solution (Sigma-Aldrich, USA) for 1 h. The following primary and secondary antibodies were used for immunostaining: Ki-67 (Cell Signalling Technology, USA or Milteny Biotec, Germany); H3K9me3 (Active Motif, USA); Donkey Anti-Mouse IgG H&L (Alexa Fluor® 488) (Abcam, Great Britain); Goat Anti-Rabbit IgG H&L (Alexa Fluor® 568) (Abcam, Great Britain); Goat anti-Rabbit IgG (H+L) (PE-Alexa Fluor™ 647) (Invitrogen, USA). Hoechst 33342 (Invitrogen, USA) was used for nuclear staining. The samples were analyzed using the Olympus BX 51 fluorescence microscope (Olympus Corporation, Japan) and Nikon A1 scanning laser confocal microscope (Nikon Corporation, Japan).

### Gene expression analysis using real-time PCR

The Rizol reagent (diaGene, Russia) was used for RNA isolation following the manufacturer’s protocol. The complementary DNA was synthesized with reverse transcription reagent kit (Biolabmix, Russia). The realtime PCR was performed using BioMaster UDG HS- qPCR SYBR Blue premixes (Biolabmix, Russia). The *ACTB* and *SDHA* were used as reference genes for normalization.

Below are the sequences of oligonucleotides used in our work:

ACTB_F ACAGAGCCTCGCCTTTG, ACTB_ RCCTTGCACATGCCGGAG;

SDHA_F TTTGATGCAGTGGTGGTAGG, SDHA_R CAGAGCAGCATTGATTCCTC;

p21_F TGGAGACTC TCAGGGTCGAAA, p21_R GGCGTTTGGAGTG GTAGAAATC;

HMGB2_F CTTGGCACGATATGCAGCAA, HMGB2_R CAGCCAAAGATAAACAACCATATGA;

LMNB1_F ACACTTCTGAACAGGATCAACC, LMNB1_R CTGTGACACCAGCGTTTGC;

p16ink4a_F CCCAACGCACCGAATAGTTA, p16ink4a_R ACCAGCGTGTCCAGGAAG;

IL6_F GTGGCTGCAGGACATGACAA, IL6_R TGA GGTGCCCATGCTACATTT;

IL8_F AAGAGCCAGGAAGAAACCACC, IL8_R CTGCAGAAATCAGGAAGGCTG;

IL1b_F CTGTCCTGCGTGTTGAAAGA, IL1b_R TTGGGTAATTTTTGGGATCTACA;

PAI1-F CTCATCAGCCACTGGAAAGGCA, PAI1-R GACTCGTGAAGTCAGCCTGAAAC;

MCP1_F CTTCTGTGCCTGCTGCTCATA, MCP1_R CTTTGGGACACTTGCTGCTG;

MMP1-F TGGACGTTCCCAAAATCCTG, MMP1-R AAGGGATTTGTGCGCATGTAG;

MMP3-F CTGCTGTTGAGAAAGCTCTG, MMP3-R AATTGGTCCCTGTTGTATCCT.

### Measurement of the telomere length using real-time PCR

The real-time PCR was performed with BioMaster UDG HS-qPCR SYBR Blue premixes (Biolabmix, Russia) using primer pairs Tel-F CGGTTTGT TTGGGTTTGGGTTTGGGTTTGGGTTTGGGTT and Tel- RGGCTTGCCTTACCCTTACCCTTACCCTTACCCTTA CCCT specific to telomeric DNA repetitive sequence as well as 36B4u CAGCAAGTGGGAAGGTGTAATCC and 36B4d CCCATTCTATCATCAACGGGTACAA specific to the region of acidic ribosomal phosphoprotein P0 gene on chromosome 12. Genome copy numbers and the total length of the telomeric DNA were determined relative to the DNA strand, represented by the plasmid pAL2-T (Eurogene, Russia) bearing 36B4 genomic region or human telomeric DNA fragment encompassing 14 repetitive units with the total length of 84 bp. The following parameters of amplification were used: 50ºС for 5 min, 95ºС for 10 min, 39 cycles of 95ºС for 15 s and 60ºС for 1 min.

### Analysis of the next-generation transcriptomic sequencing data and building predictive models based on the transcriptome data

The datasets deposited in the Gene Expression Omnibus repository (GSE139073, GSE145008) were used in our work [23, 24]. Short-read alignment to the reference GRCh38 genome, preprocessing, and detection of gene expression were performed using STAR, SAMtools, and featureCounts programs [25–27]. To eliminate batch effects in the transcriptome data, the ComBat-seq method implemented in the sva package was applied [28]. The statistical edgeR package was employed to analyze the differential gene expression [29].

The predictive models of chronological age and *in vitro* cultivation duration were created based on the normalized values of gene expression. Genes, whose expression level correlated significantly with the passage or chronological age of the donors, were selected using Spearman and Pearson coefficients of correlation (coefficient >|0.5|, p-value-adjusted <0.05). The regressive models were built using LASSO regression and random forest regressor (RFR) from the Scikit-learn package [30]. Data were divided into two sets: the training set (80%, 84 sequencing samples) and the test set (20%, 22 sequencing samples). For the LASSO regressionbased model, automatic tuning of the hyperparameter was applied with LassoCV on the training set. For the RFR model, the base number of tree parameters was used. The model quality was evaluated on the test set which was not involved in the learning process.

### Data preparation and training of the neural network segmentation model

In the first stage, using the segment anything image-recognition model followed by manual validation and correction, nuclear masks were generated for microscopic images of cell preparations of umbilical MSCs at different culture passages (passage range — 3–15, a total of 27,500 cells) [31]. At the next stage, images were scaled to the equal resolution and divided by a sliding window with a 246-pixel pitch into the overlapping fragments 256×256 in size. The window step provided an overlap of neighboring image fragments by 10 pixels on each side, which reduced boundary artifacts during the subsequent assembly of the final segmentation map. To increase the model’s robustness to various exposure and contrast variations of the images, augmentation methods were employed. Among the transformations used were horizontal and vertical flips, random adjustments of brightness and contrast, as well as scaling with small shifts. The final dataset comprised 563 examples and was split in an 80 to 20% ratio for training and testing, providing a sufficient number of samples for proper tuning of the network parameters.

For solving the cell nucleus segmentation task, a convolutional neural network architecture, DeepLabV3+ [32] was used. As the backbone network, EfficientNet-b0 [33] pretrained on the ImageNet dataset was selected, providing the models with an initial representation of low-level image features. Training was performed for 40 epochs, allowing the model to reach stable convergence. During this period, the model was trained on a compute node equipped with an NVIDIA A100 GPU, completing the full training cycle in 3.5 h. To minimize the impact of class imbalance (significant differences in nucleus sizes and thin boundaries) and achieve more accurate segmentation, a combined loss function was used, which integrated two components: the BCE-Dice Loss provided high sensitivity to the imbalance between classes (nucleus/background) and accounted for spatial consistency of predictions; the focal loss improved training by reducing the influence of easily classified examples. During training, a learning-rate scheduler was employed, adjusting the learning rate from an initial value of 1e–3 down to 1e–5 after each iteration, ensuring a gradual reduction of the optimization step and promoting stable convergence of the model.

For calculating quantitative morphometric characteristics of nuclei, a binary mask obtained from the DeepLabV3+ segmentation results was passed to the analysis function. Before the calculations, pixels marked as “border” were excluded from the overall mask, after which sequential erosion and dilation (by 20 pixels) were performed to remove thin artifacts and merge broken contours. The parameters computed for each nucleus: center coordinates (X, Y), area, roundness, semi-major/semi-minor axes of the ellipse and inclination angle, the Hausdorff distance. The coefficient of belonging to the class was also established: class 1 (passages 3–5), class 2 (passages 7–9), class 3 (passages 11–15). The executable scripts of the model are deposited in the GitHub repository (https://github.com/LabADTCellSeg/cellseg).

## Results

### Senescence-associated changes in gene expression profile

To assess the senescence- associated gene expression dynamics in MSCs we selected genes that might be considered as principle regulators of the cell cycle and nucleus structure, as well as genes encoding components of the proinflammatory phenotype. We examined the expression levels of the following genes — *P16INK4a*/*СDKN2A*, *P21CIP1*/ *CDKN1A*, *LMNB1*, *HMGB2*, *IL6*, *IL8*/*CXCL8*, *IL1B*, *SERPINE1/PAI1*, *MCP1*/*CCL2*, *MMP1*, *MMP3*. Expression analysis was performed on a replicative senescence model: independent umbilical cord MSCs subjected to long-term culture (n=2); on a chronological aging model: BM-MSCs from the donors of different ages: 20–25 years (n=2) and older than 65 years (n=2); on the model of stress-induced cellular senescence: umbilical cord MSCs exposed to hydrogen peroxide to induce cellular senescence (n=2).

**Figure 1.**
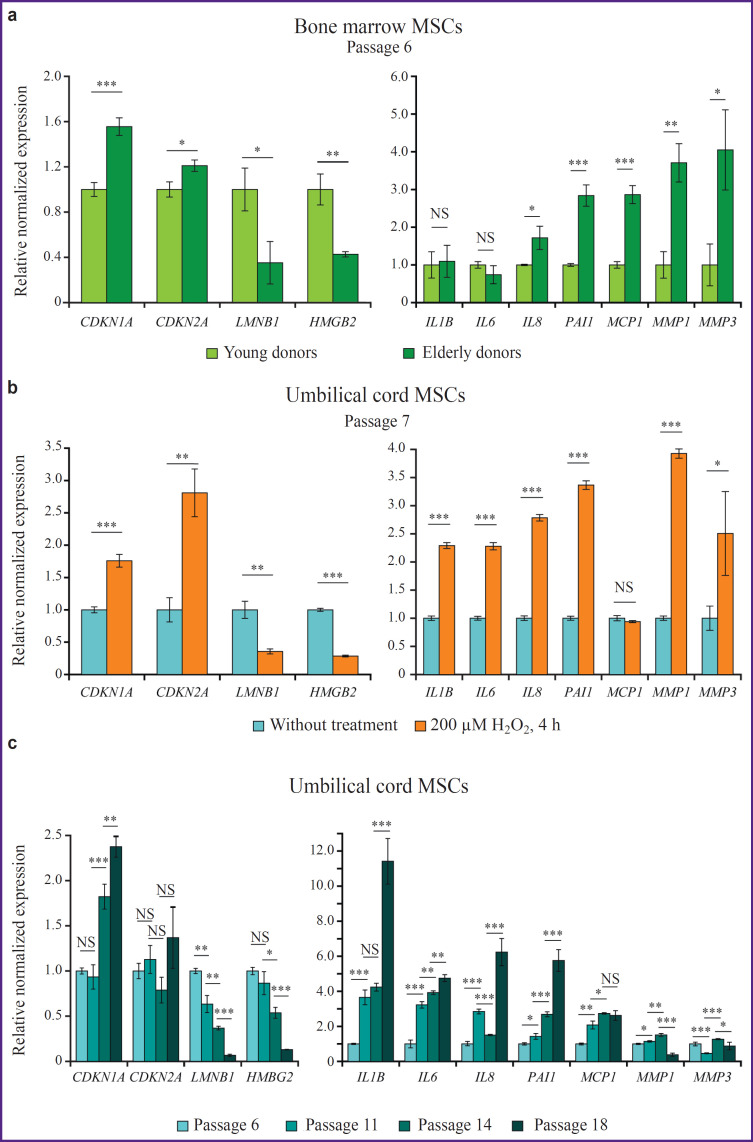
Relative normalized gene expression measured by RT-qPCR in the MSC cultures: (a) comparison of gene expression in the cultures of MSCs (passage 6) derived from young donors (18–25 years, n=3) and elderly donors (>65 years, n=3); (b) comparison between the umbilical MSCs (passage 7) cultured under standard conditions and after 4-hour treatment of 200 μM solution of hydrogen peroxide; (c) gene expression dynamics during cultivation of umbilical MSCs under standard conditions, measurements taken at passages 6, 11, 14, and 18 are presented; * p<0.05; ** p<0.005; *** p<0.0005; NS — p≥0.05, Student’s t-test

The genes encoding cyclin-dependent kinase inhibitors (*СDKN2A* and *CDKN1A*) displayed a similar dynamics across all examined samples. Their expression increased during prolonged culture, in response to oxidative stress and with increasing donor age (Figure 1). Notably, the increase of expression was more pronounced for *CDKN1A*, whereas changes of *CDKN2A* expression were weaker and, in the context of the replicative aging, insignificant. The expression level of the genes encoding the nuclear architectural proteins LMNB1 and HMGB2 consistently decreased both in replicative and stress-induced senescence models as well as during chronological MSC aging (see Figure 1).

The most dramatic changes were observed during replicative senescence, where the decline in expression over the culture period reached about 90% for both *LMNB1* and *HMGB2*. It is noteworthy that genes encoding the SASP components exhibited different dynamics depending on the cause of cellular senescence (see Figure 1). For example, peroxideinduced senescence led to an increase in the expression levels of all studied genes except *MCP1*, whereas replicative senescence did not activate the genes encoding matrix metalloproteinases MMP1 and MMP3. It is also worth noting that analysis of the BM-MSCs from the donors of different ages did not reveal age-related changes at the expression levels of the cytokine genes *IL-6*, *IL-1β*, *CXCL8* was weakly activated in the cells of the elderly donors. The *SERPINE1/PAI1* gene, encoding PAI-1 protein, appeared to be most stable in the context of aging-associated dynamics of gene expression.

Various manifestations of senescence-associated proinflammatory phenotype depending on the type of cellular senescence are generally expected. Nevertheless, to independently verify the obtained results, we searched for the publicly available high- throughput transcriptome sequencing datasets of cultured MSCs that include donor age and cell passage information in the Gene Expression Omnibus repository. As a result, a dataset (n=37, age 3–85 years, median value 47 years; Table 1) has been prepared and correlation analysis of expression changes in the tested genes with prolonged cultivation and donor age has been performed. According to the obtained results, *SERPINE1/PAI1*, *CDKN1A*, and *CDKN2A* genes demonstrated a positive correlation with culture duration, whereas *LMNB1* and *HMGB2* showed a negative correlation (Spearman correlation, p<0.05; Table 2). The correlation with the donor age was detected for *CDKN1A* and *LMNB1* genes when no correction for multiple testing was applied (Table 3). Differential expression analysis in BM-MSC samples from donors aged 20–35 years (n=7) and donors aged 60–85 years (n=13) has identified only 50 differentially expressed genes (|logFC|>2, p<0.05) (Table 4). It is highly probable that donor-dependent variability of gene expression patterns in MSCs can be quite substantial, making it difficult to identify transcriptional markers of chronological aging. Nevertheless, the expression of individual genes might be applicable for assessing cellular senescence *in vitro*.

**T a b l e 1:** The list of the RNA-seq datasets used in the study

Sample identifier	Donor age	Gender	Passage	Donor identification number
***Project identifier — GSE139073***				
SRR10307337	73	F	4	777
SRR10307338	73	F	4	777
SRR10307339	48	F	4	819
SRR10307340	48	F	4	819
SRR10307341	75	F	4	821
SRR10307342	75	F	4	821
SRR10307343	24	M	3	126
SRR10307344	24	M	3	126
SRR10307345	16	F	3	127
SRR10307346	16	F	3	127
SRR10307347	61	M	3	237
SRR10307348	61	M	3	237
SRR10307349	25	F	3	264
SRR10307350	25	F	3	264
SRR10307351	63	M	3	265
SRR10307352	63	M	3	265
SRR10307353	48	F	3	276
SRR10307354	48	F	3	276
SRR10307355	82	F	3	278
SRR10307356	82	F	3	278
SRR10307357	35	F	3	285
SRR10307358	35	F	3	285
SRR10307359	45	F	3	289
SRR10307360	45	F	3	289
SRR10307361	48	M	3	293
SRR10307362	48	M	3	293
SRR10307363	47	F	3	308
SRR10307364	47	F	3	308
SRR10307365	71	F	3	316
SRR10307366	71	F	3	316
SRR10307367	51	M	3	324
SRR10307368	51	M	3	324
SRR10307369	57	M	3	329
SRR10307370	57	M	3	329
SRR10307371	80	M	3	336
SRR10307372	80	M	3	336
SRR10307373	85	M	3	354
SRR10307374	85	M	3	354
SRR10307375	37	M	3	357
SRR10307376	37	M	3	357
SRR10307377	68	M	3	374
SRR10307378	68	M	3	374
SRR10307379	78	M	3	378
SRR10307380	78	M	3	378
SRR10307381	68	F	3	386
SRR10307382	68	F	3	386
SRR10307383	65	F	3	660
SRR10307384	65	F	3	660
SRR10307385	69	F	3	651
SRR10307386	69	F	3	651
SRR10307387	73	F	3	777
SRR10307388	73	F	3	777
SRR10307389	33	M	3	784
SRR10307390	33	M	3	784
SRR10307391	24	M	6	126
SRR10307392	24	M	6	126
SRR10307393	16	F	6	127
SRR10307394	16	F	6	127
SRR10307395	35	F	6	285
SRR10307396	35	F	6	285
SRR10307397	48	M	6	293
SRR10307398	48	M	6	293
SRR10307399	51	M	6	324
SRR10307400	51	M	6	324
SRR10307401	33	M	6	784
SRR10307402	33	M	6	784
***Project identifier — GSE145008***				
SRR11050732	14	F	3	1
SRR11050733	14	F	3	1
SRR11050734	14	F	3	1
SRR11050735	14	F	3	1
SRR11050736	20	M	3	2
SRR11050737	20	M	3	2
SRR11050738	20	M	3	2
SRR11050739	20	M	3	2
SRR11050740	9	F	3	3
SRR11050741	9	F	3	3
SRR11050742	9	F	3	3
SRR11050743	9	F	3	3
SRR11050744	5	M	3	4
SRR11050745	5	M	3	4
SRR11050746	9	F	3	5
SRR11050747	9	F	3	5
SRR11050748	9	F	3	5
SRR11050749	9	F	3	5
SRR11050750	13	F	3	6
SRR11050751	13	F	3	6
SRR11050752	13	F	3	6
SRR11050753	13	F	3	6
SRR11050754	29	M	3	7
SRR11050755	29	M	3	7
SRR11050756	29	M	3	7
SRR11050757	29	M	3	7
SRR11050758	17	M	3	8
SRR11050759	17	M	3	8
SRR11050760	33	F	3	9
SRR11050761	33	F	3	9
SRR11050762	33	F	3	9
SRR11050763	33	F	3	9
SRR11050764	13	F	3	10
SRR11050765	13	F	3	10
SRR11050766	13	F	3	10
SRR11050767	13	F	3	10
SRR11050768	3	F	3	11
SRR11050769	3	F	3	11
SRR11050770	3	F	3	11
SRR11050771	3	F	3	11

**T a b l e 2:** Correlation of expression of gene markers of cell senescence with the duration of bone marrow MSC cultivation according to transcriptomic data

Gene	Spearman correlation coefficient	p-value	p-value corrected by the Benjamini–Hochberg method
*SERPINE1/PAI1*	0.45	1.04E–06	0.000053
*HMGB2*	–0.33	0.00049	0.0052
*LMNB1*	–0.31	0.0013	0.011
*CDKN1A*	0.31	0.0015	0.012
*CDKN2A*	0.30	0.0017	0.014
*CXCL8*	0.25	0.011	0.054
*CCL2*	0.22	0.023	0.090
*IL6*	0.22	0.024	0.091
*IL1B*	0.12	0.22	0.44
*MMP3*	–0.10	0.29	0.53

**T a b l e 3:** Correlation of expression of gene markers of cell senescence with the age of bone marrow MSCs donors according to transcriptomic data

Gene	Spearman correlation coefficient	p-value	p-value corrected by the Benjamini–Hochberg method
*CDKN1A*	0.21	0.027	0.14
*LMNB1*	–0.21	0.032	0.16
*HMGB2*	–0.19	0.050	0.21
*MMP3*	0.17	0.078	0.27
*CXCL8*	0.17	0.086	0.28
*SERPINE1/PAI1*	0.11	0.27	0.53
*CCL2*	0.10	0.29	0.54
*IL6*	0.045	0.65	0.82
*IL1B*	–0.024	0.80	0.91
*CDKN2A*	–0.014	0.89	0.95

**T a b l e 4:** Differentially expressed genes in bone marrow MSC RNA-seq samples from donors aged 20–35 years and donors aged 60–85 years

Gene	logFC	logCPM	LR	p-value	FDR
*IDO1*	–6.74201	0.130015	17.82139	2.43E–05	0.010439
*IGKC*	–6.03135	1.491429	33.98931	5.54E–09	4.51E–05
*CSF3*	–6.00031	–0.9205	13.5434	0.00023311	0.038058
*CXCL9*	–5.91117	–0.96128	13.78282	0.000205204	0.035238
*IGHG1*	–5.75566	–0.59634	36.87759	1.26E–09	2.66E–05
*IGLC2*	–5.718	–1.29152	36.70287	1.38E–09	2.66E–05
*EEF1DP5*	–5.41503	–0.55565	13.93917	0.000188822	0.034148
*IGHA1*	–5.19498	–1.61628	29.47482	5.66E–08	0.000181
*IGKV4-1*	–4.98465	–1.7487	30.30424	3.69E–08	0.000151
*MYOD1*	–4.95005	–1.06079	20.98677	4.62E–06	0.004005
*MUC5AC*	–4.60036	–1.35788	14.91265	0.000112605	0.02448
*IGHG3*	–4.53654	–1.75797	22.59954	2.00E–06	0.002714
*IGLC3*	–4.45419	–1.9868	20.78149	5.15E–06	0.004046
*IGHG2*	–4.45175	–1.99772	21.74035	3.12E–06	0.003496
*GBX2*	–4.38756	–1.50842	27.1453	1.89E–07	0.000514
*IGLV3-19*	–4.23956	–2.06218	20.88334	4.88E–06	0.004005
*IGLV3-21*	–4.16984	–2.11667	21.59141	3.37E–06	0.003535
*IGLV2-14*	–3.91505	–2.21161	19.31244	1.11E–05	0.006177
*IGHM*	–3.60855	–1.94161	13.95801	0.000186939	0.033957
*MYOG*	–3.60479	–1.94571	13.91752	0.000191009	0.034342
*LAIR1*	–3.57773	–2.32914	24.23182	8.54E–07	0.001396
*TMEM176B*	–3.57146	–0.45584	22.1179	2.56E–06	0.003122
*ALKAL1*	–3.54348	–2.1191	18.05065	2.15E–05	0.00947
*IGLV2-11*	–3.49343	–2.32861	16.19594	5.71E–05	0.016806
*IGKV3-20*	–3.40265	–2.3715	15.44576	8.49E–05	0.021421
*GCGR*	–3.23964	–2.16184	17.18263	3.40E–05	0.01235
*IGKV3-15*	–3.19646	–2.42303	17.2754	3.23E–05	0.012124
*ABO*	–3.19078	–1.62966	25.39491	4.67E–07	0.000909
*SHD*	–3.17845	–1.93949	13.36813	0.000255936	0.039473
*TNMD*	–3.12462	–1.51833	26.04848	3.33E–07	0.000846
*MTND1P23*	–3.10661	–0.32258	14.56727	0.000135243	0.027729
*IGLV2-23*	–2.973	–2.26645	12.53699	0.000398975	0.049474
*CLDN5*	–2.81786	–2.22574	12.53175	0.000400096	0.049474
*IGLV6-57*	–2.75739	–2.51936	15.10868	0.000101495	0.023174
*KRTAP7-1*	–2.70262	–2.40336	15.37327	8.82E–05	0.021723
*ZFP42*	–2.62331	–2.1215	13.22307	0.000276524	0.041179
*SCN5A*	–2.5801	–1.27536	33.64228	6.62E–09	4.51E–05
*NPPB*	–2.43186	–1.75835	15.13791	9.99E–05	0.022947
*LINC01012*	–2.32618	–2.10984	25.94058	3.52E–07	0.000846
*KLHL34*	–2.29995	–2.32066	13.33429	0.000260597	0.039816
*CYP19A1*	–2.27558	–2.59913	14.27137	0.000158254	0.031096
*CACNA1S*	–2.24875	–2.60108	13.49961	0.000238613	0.038058
*FAM181B*	–2.20795	–1.61487	21.35758	3.81E–06	0.003709
*TREML3P*	–2.20459	–1.64722	18.8168	1.44E–05	0.007261
*SLC51B*	–2.08324	–2.08956	20.25594	6.77E–06	0.004944
*TRIM72*	–2.07456	–2.22947	14.87855	0.000114659	0.024664
*HOXC12*	–2.02755	0.297099	25.10952	5.42E–07	0.000963
*GPX1P2*	–2.02304	–2.63327	12.67682	0.000370216	0.048132
*LINC03004*	–2.00501	–1.67803	13.22407	0.000276377	0.041179
*CRTAC1*	–1.98816	–1.90343	24.45347	7.61E–07	0.001296
*GPR83*	–1.95678	–2.02969	19.58557	9.62E–06	0.006084
*NKX2-2*	–1.95335	–1.98009	15.052	0.000104589	0.023548
*PDE1B*	–1.93211	–2.01438	13.0485	0.000303527	0.04321
*HEY2-AS1*	–1.89023	–2.0207	12.65258	0.000375047	0.048415
*NOTCH4*	–1.88533	–2.50867	12.50129	0.00040667	0.049615
*MYH14*	–1.88023	0.589392	15.27985	9.27E–05	0.022663
*ZNF728*	–1.87424	–2.18341	14.43592	0.00014501	0.02877
*ZNF99*	–1.8592	–2.06605	19.20551	1.17E–05	0.00634
*WNK2*	–1.80691	–2.04595	16.79481	4.16E–05	0.013727
*RSAD2*	–1.79102	–1.69653	12.85613	0.000336376	0.046134
*DUSP15*	–1.73147	–0.94925	22.6271	1.97E–06	0.002714
*SUNO1*	–1.73101	–1.89769	16.7206	4.33E–05	0.013937
*GIPC3*	–1.70111	–1.47442	35.46025	2.60E–09	2.66E–05
*XIRP1*	–1.70079	–0.95231	22.31142	2.32E–06	0.002961
*LINC02182*	–1.70015	–1.38832	32.94003	9.50E–09	4.86E–05
*PCSK1N*	–1.69436	–0.279	35.46079	2.60E–09	2.66E–05
*RBM12B-DT*	–1.68636	–2.31889	13.81687	0.000201519	0.035154
*APCDD1*	–1.6716	–0.15046	22.09332	2.60E–06	0.003122
*TMEM63C*	–1.65885	–1.67849	14.07606	0.000175564	0.032973
*DUSP26*	–1.65032	–1.58372	17.78676	2.47E–05	0.01052
*GDF10*	–1.6433	–0.98772	16.74222	4.28E–05	0.013937
*PHLDA2*	–1.6418	0.535531	15.23313	9.50E–05	0.022711
*LTK*	–1.63865	–1.58262	21.49046	3.56E–06	0.003627
*LINC00937*	–1.59819	–2.06279	19.78999	8.64E–06	0.005791
*LHX4*	–1.59619	–2.04904	15.95387	6.49E–05	0.017804
*TRIM67*	–1.59194	–0.12995	13.15763	0.000286351	0.041865
*PLXDC1*	–1.56106	–1.03286	27.39377	1.66E–07	0.000485
*L1CAM*	–1.52989	0.276793	14.43591	0.00014501	0.02877
*SERPINA12*	–1.5203	–0.61139	29.44086	5.76E–08	0.000181
*SLC30A3*	–1.51219	0.315307	18.86388	1.40E–05	0.007172
*FAM162B*	–1.50741	–1.53032	15.20718	9.63E–05	0.022759
*CMPK2*	–1.50377	–1.52247	15.84972	6.86E–05	0.018562
*HSPB3*	–1.50214	–0.89656	22.53947	2.06E–06	0.002714
*TMEM191B*	–1.49286	–2.02685	13.79539	0.000203835	0.035154
*TMEM156*	–1.45208	–1.7143	12.83349	0.00034047	0.046385
*TMOD1*	–1.43636	–0.74442	21.17288	4.20E–06	0.00393
*GPAT2P1*	–1.40596	–1.70658	12.56297	0.000393465	0.049474
*GPR27*	–1.40062	0.30417	19.32474	1.10E–05	0.006177
*CDH8*	–1.39788	–0.37092	17.14553	3.46E–05	0.01235
*CACNA2D3*	–1.39769	2.11436	14.7683	0.000121562	0.025479
*HEY2*	–1.39228	2.025565	19.43099	1.04E–05	0.006177
*ST8SIA2*	–1.39093	–0.49211	17.53827	2.82E–05	0.010961
*LINC02056*	–1.38857	–1.62073	12.69264	0.000367098	0.048132
*LRP2*	–1.37985	–1.28367	23.2891	1.39E–06	0.00211
*TGFA*	–1.37091	–0.4596	25.61174	4.17E–07	0.000853
*WFDC1*	–1.36441	2.938302	15.38643	8.76E–05	0.021703
*HES4*	–1.34238	2.562402	17.64995	2.66E–05	0.010735
*PTH1R*	–1.33645	0.127042	20.95353	4.71E–06	0.004005
*SYN2*	–1.26943	1.567083	12.94207	0.000321279	0.044663
*HOXC13*	–1.26823	–0.37058	19.43103	1.04E–05	0.006177
*RAI2*	–1.26422	–0.02813	12.60263	0.000385204	0.048893
*LONRF2*	–1.21634	0.213618	16.65195	4.49E–05	0.014227
*HEYL*	–1.2162	2.019352	18.99141	1.31E–05	0.00688
*CSPG5*	–1.21002	–1.72016	18.2359	1.95E–05	0.008993
*HEY1*	–1.20268	–0.67351	13.30536	0.000264648	0.040107
*SLFN14*	–1.19595	–1.77817	13.91193	0.000191579	0.034342
*ADCY2*	–1.1846	2.069172	15.06383	0.000103936	0.023548
*RASGRP2*	–1.17855	–0.48357	14.0089	0.000181948	0.033347
*HOXC13-AS*	–1.17836	–1.28119	15.17852	9.78E–05	0.022901
*WIPF3*	–1.17172	–0.55415	19.45319	1.03E–05	0.006177
*PDZD4*	–1.16655	0.813909	17.21875	3.33E–05	0.01235
*CCDC3*	–1.16413	1.018813	15.73623	7.28E–05	0.0192
*NPTX1*	–1.15928	–1.25645	13.03203	0.000306208	0.04321
*CNTN1*	–1.15545	0.424462	12.51781	0.000403091	0.049474
*ADAP1*	–1.1469	–0.05287	25.73178	3.92E–07	0.000853
*ITIH5*	–1.13921	6.341922	13.5147	0.000236702	0.038058
*LINC00547*	–1.13275	0.376618	17.9216	2.30E–05	0.010009
*SOX18*	–1.12464	1.243256	15.23793	9.48E–05	0.022711
*LGALS9*	–1.11907	–0.08648	14.93291	0.000111402	0.024348
*LINC01362*	–1.07221	–1.14826	16.16752	5.80E–05	0.016806
*NAP1L2*	–1.06613	–1.41077	18.36179	1.83E–05	0.008698
*SLC24A3*	–1.06481	3.570396	14.93962	0.000111007	0.024348
*KNDC1*	–1.04924	–0.4902	12.71091	0.000363528	0.048132
*TMEM151A*	–1.04248	0.0769	13.21936	0.000277072	0.041179
*CX3CL1*	–1.0329	–0.55568	19.79463	8.62E–06	0.005791
*GNAO1*	–1.02459	0.68505	12.50729	0.000405366	0.049604
*KCNB1*	–1.00315	1.293309	13.64308	0.000221055	0.036797
*KL*	–0.99468	–1.5026	12.69037	0.000367543	0.048132
*CDH15*	–0.96785	3.09543	21.71385	3.16E–06	0.003496
*FGFR3*	–0.96435	1.855654	18.18702	2.00E–05	0.008993
*RTN1*	–0.95672	–0.85915	13.51578	0.000236566	0.038058
*SORBS1*	–0.91465	0.58739	12.917	0.000325611	0.045108
*GPRC5C*	–0.91125	1.346111	17.15483	3.45E–05	0.01235
*FOLR1*	–0.90256	0.48096	12.62354	0.000380919	0.048707
*ADCY1*	–0.89133	0.016148	16.82263	4.10E–05	0.013637
*CD247*	–0.88122	–0.95038	20.89899	4.84E–06	0.004005
*PHOSPHO1*	–0.87691	–1.10453	19.19722	1.18E–05	0.00634
*RNASEK*	–0.8769	–1.24948	15.80022	7.04E–05	0.018682
*LRRC3*	–0.87242	2.006731	17.69325	2.60E–05	0.010735
*ACAN*	–0.87036	10.49467	19.38983	1.07E–05	0.006177
*WDR87BP*	–0.86428	0.707841	14.02695	0.000180209	0.033327
*AKAP6*	–0.85959	2.440635	18.39394	1.80E–05	0.008698
*TSPAN15*	–0.85178	2.428343	15.81195	7.00E–05	0.018682
*CARMIL2*	–0.83114	–0.62056	12.58457	0.000388945	0.049215
*FAM83H*	–0.80075	–0.53574	19.45025	1.03E–05	0.006177
*MARK1*	–0.79935	1.851002	17.57187	2.77E–05	0.010872
*TPD52*	–0.79811	0.520582	15.93241	6.56E–05	0.017887
*DYSF*	–0.79424	5.192088	14.96679	0.00010942	0.0243
*FAM169A*	–0.77762	–0.93632	13.44043	0.00024626	0.038563
*NOG*	–0.77472	3.337352	13.13857	0.000289278	0.042065
*ADRA1B*	–0.77342	–0.31774	12.53894	0.000398558	0.049474
*SHANK2*	–0.76822	2.303994	13.88607	0.000194233	0.034666
*BRSK2*	–0.74595	0.213741	13.98842	0.00018394	0.033562
*DNAH10*	–0.73965	–0.88267	13.19353	0.000280917	0.041599
*RYR1*	–0.73799	–0.41911	13.08336	0.00029793	0.042876
*LEPR*	–0.73782	8.314245	13.50392	0.000238066	0.038058
*SHC4*	–0.73431	1.564619	15.21512	9.59E–05	0.022759
*GIPR*	–0.73217	0.662796	15.49546	8.27E–05	0.021049
*ENPEP*	–0.71631	1.032989	14.88719	0.000114135	0.024664
*LINC02600*	–0.71439	–0.12262	13.67039	0.000217862	0.036643
*RASL11A*	–0.70716	2.188546	14.50159	0.000140041	0.028335
*NPTXR*	–0.707	5.032025	13.53486	0.000234173	0.038058
*TMEM54*	–0.69739	0.126718	13.38037	0.000254272	0.039365
*FOXCUT*	–0.694	0.89011	16.11985	5.95E–05	0.017113
*ARHGEF16*	–0.68756	1.494923	12.51938	0.000402753	0.049474
*FAM89A*	–0.68137	3.487588	15.6699	7.54E–05	0.019758
*GPAT2*	–0.67632	1.848891	14.75442	0.00012246	0.025536
*ITGAL*	–0.6678	0.188307	15.82416	6.95E–05	0.018682
*IGFBP2*	–0.66553	8.122881	13.76376	0.000207297	0.035238
*CASQ1*	–0.65423	–0.07883	13.49412	0.000239312	0.038058
*IL7R*	–0.64593	4.366858	13.44428	0.000245754	0.038563
*SLC16A14*	–0.6331	0.173104	13.79726	0.000203632	0.035154
*MICA*	–0.6268	2.579888	17.04439	3.65E–05	0.012756
*GP1BB*	–0.614	2.047757	15.41856	8.61E–05	0.021598
*DGKG*	–0.61299	1.438626	14.59997	0.000132917	0.027437
*JPH2*	–0.60239	5.92529	13.87562	0.000195316	0.0347
*EDN1*	–0.59572	2.794714	12.7033	0.00036501	0.048132
*CCDC158*	–0.5949	1.517501	14.05138	0.000177883	0.033047
*ADRA2C*	–0.59458	3.684148	12.98359	0.000314233	0.043983
*EGFL7*	–0.59008	3.282763	14.19471	0.000164834	0.032081
*ITGB1BP2*	–0.58525	–0.13723	13.22001	0.000276977	0.041179
*LYL1*	–0.58504	0.516555	12.62508	0.000380605	0.048707
*ZSWIM5*	–0.5718	0.176161	15.03511	0.000105529	0.023569
*QPRT*	–0.57054	1.791185	20.97442	4.65E–06	0.004005
*CKB*	–0.54894	6.4886	21.15728	4.23E–06	0.00393
*SYNGR2*	–0.54869	4.063023	17.63299	2.68E–05	0.010735
*C3orf70*	–0.53617	2.258994	14.40221	0.000147629	0.029148
*SEPTIN5*	–0.53548	5.850167	17.71423	2.57E–05	0.010735
*LGMN*	–0.5295	7.390066	13.44756	0.000245325	0.038563
*CRIP1*	–0.5278	5.915864	15.04709	0.000104862	0.023548
*LINC00702*	–0.52483	1.68425	18.78584	1.46E–05	0.007289
*SLC37A1*	–0.51686	2.285977	15.25594	9.39E–05	0.022704
*SRRM3*	–0.50967	1.020992	12.7151	0.000362716	0.048132
*ZNF469*	–0.50237	6.320352	21.44639	3.64E–06	0.003627
*CRYAB*	–0.50215	8.082892	18.24895	1.94E–05	0.008993
*DNAJC6*	–0.50021	3.746198	16.17961	5.76E–05	0.016806
*PPFIA3*	–0.49208	1.786657	18.40639	1.78E–05	0.008698
*CGREF1*	–0.48846	3.916935	15.49059	8.29E–05	0.021049
*DNAH5*	–0.48696	1.114283	14.10597	0.000172794	0.032848
*HES6*	–0.48063	1.574922	20.41189	6.24E–06	0.00464
*CSPG4*	–0.47527	7.532437	13.59239	0.000227104	0.037427
*IRAG1*	–0.45697	4.251489	12.48452	0.000410338	0.04986
*TBXA2R*	–0.45102	2.367274	17.31301	3.17E–05	0.011997
*HS6ST1*	–0.44571	5.327054	13.75934	0.000207785	0.035238
*DMPK*	–0.44482	6.388112	29.74076	4.94E–08	0.000181
*LYSMD2*	–0.41007	1.900144	13.60586	0.00022548	0.03731
*ANKRD9*	–0.40992	4.467814	17.67927	2.61E–05	0.010735
*NOTCH3*	–0.40918	9.646443	15.15878	9.88E–05	0.022945
*ISG20*	–0.39744	2.672711	13.30319	0.000264954	0.040107
*ADORA2B*	–0.39485	2.24752	14.86052	0.000115761	0.024756
*GPC4*	–0.39132	6.164633	16.08634	6.05E–05	0.017248
*HSPB1*	–0.38434	9.410244	22.73045	1.86E–06	0.002714
*CYB5R1*	–0.38118	5.770637	15.1487	9.94E–05	0.022945
*SLC2A6*	–0.3756	4.189715	15.40244	8.69E–05	0.021651
*MSRB1*	–0.3701	5.511516	14.55164	0.00013637	0.027729
*ENDOD1*	–0.36543	6.610153	14.06412	0.000176682	0.032973
*SIX2*	–0.36102	5.707384	13.44129	0.000246147	0.038563
*RAVER2*	–0.35903	4.2544	19.80343	8.58E–06	0.005791
*TUBB2A*	–0.34554	5.187744	14.4532	0.000143686	0.02877
*MIEN1*	–0.34286	1.951609	13.04021	0.000304874	0.04321
*RGS19*	–0.33713	3.360489	12.52686	0.000401143	0.049474
*PSEN2*	–0.33481	4.018959	19.30059	1.12E–05	0.006177
*GSN*	–0.32799	9.22319	21.84951	2.95E–06	0.003444
*MAP2K3*	–0.32026	6.621549	25.12837	5.36E–07	0.000963
*MGAT5*	–0.30153	6.814577	15.61065	7.78E–05	0.020257
*SNTA1*	–0.29562	5.384169	12.61557	0.000382546	0.048707
*PTPN3*	–0.29194	3.537507	18.04734	2.15E–05	0.00947
*SORT1*	–0.29142	7.62097	16.69438	4.39E–05	0.014021
*SRD5A1*	–0.28284	4.729072	25.68423	4.02E–07	0.000853
*TPST2*	–0.28145	6.976341	18.20642	1.98E–05	0.008993
*CALM1*	–0.27529	8.260706	17.00462	3.73E–05	0.012807
*ROGDI*	–0.27022	4.824984	15.59218	7.86E–05	0.020327
*DAB2IP*	–0.26752	5.951331	17.01764	3.70E–05	0.012807
*DIRAS1*	–0.2544	5.512156	12.89333	0.000329755	0.045378
*FAM219A*	–0.25096	5.24701	21.62318	3.32E–06	0.003535
*EHD1*	–0.24981	7.699704	23.91399	1.01E–06	0.001584
*BCAP31*	–0.24721	7.121209	13.18563	0.000282103	0.041624
*CTNNB1*	–0.24353	8.746768	16.89087	3.96E–05	0.013263
*RHOC*	–0.23978	8.846199	13.7611	0.000207591	0.035238
*HDAC5*	–0.23914	5.900239	15.55691	8.01E–05	0.020579
*INPP5A*	–0.23369	5.185924	12.83613	0.00033999	0.046385
*CUEDC1*	–0.23047	6.109555	17.66143	2.64E–05	0.010735
*LASP1*	–0.22691	9.744295	18.35837	1.83E–05	0.008698
*FEZ2*	–0.22165	6.185835	13.57329	0.000229427	0.037658
*IGHMBP2*	–0.22138	4.300589	14.06617	0.00017649	0.032973
*EMP3*	–0.22047	7.715333	12.61791	0.000382068	0.048707
*PREB*	–0.2187	5.839774	12.72631	0.000360548	0.048132
*DDRGK1*	–0.21732	5.515303	15.17375	9.81E–05	0.022901
*HDAC11*	–0.20866	4.352068	17.601	2.72E–05	0.010811
*PITPNM1*	–0.20846	5.371332	13.86789	0.000196121	0.0347
*LDLRAP1*	–0.20678	5.838397	13.05207	0.000302949	0.04321
*ARHGEF10L*	–0.20518	5.919073	12.77922	0.000350491	0.04727
*NPTN*	–0.20116	8.061508	14.95693	0.000109993	0.0243
*SNX11*	–0.20075	4.663461	17.13849	3.48E–05	0.01235
*NAPA*	–0.19531	4.064688	14.61631	0.000131769	0.027338
*TMEM109*	–0.19072	6.596263	14.83157	0.000117551	0.024893
*SPRYD3*	–0.18655	6.312584	12.77161	0.000351921	0.04727
*MAPRE3*	–0.17976	4.672064	12.48096	0.000411121	0.04986
*KIF1C*	–0.17932	8.266324	19.72121	8.96E–06	0.005907
*PPP2CB*	–0.17822	6.651028	16.61981	4.57E–05	0.014249
*RHBDD2*	–0.17806	5.673687	19.5745	9.68E–06	0.006084
*SELENOS*	–0.17695	6.242757	13.12572	0.00029127	0.042065
*SLC27A4*	–0.17567	5.214289	12.51806	0.000403037	0.049474
*ARMCX3*	–0.17348	6.995281	13.15464	0.000286808	0.041865
*RMDN3*	–0.16785	5.427733	13.50392	0.000238065	0.038058
*LRRFIP2*	–0.15981	6.355986	13.13037	0.000290548	0.042065
*PPP2R1A*	–0.15488	7.886332	12.99828	0.000311777	0.043789
*SEC14L1*	–0.15293	6.814728	15.27059	9.32E–05	0.022663
*EHBP1L1*	–0.14069	7.671404	14.1189	0.000171611	0.032848
*BLCAP*	–0.13762	6.3339	13.40166	0.000251401	0.039069
*LZTS2*	–0.13014	6.888952	12.78966	0.000348541	0.04727
*GART*	0.144891	5.426869	16.73461	4.30E–05	0.013937
*NUDT21*	0.146174	5.944158	12.67975	0.000369637	0.048132
*PTGR3*	0.151359	4.48144	12.68544	0.000368514	0.048132
*HEATR6*	0.168375	4.60623	16.04382	6.19E–05	0.017446
*PSIP1*	0.174873	5.351493	12.7672	0.00035275	0.04727
*CMTR2*	0.176201	4.585269	17.05302	3.64E–05	0.012756
*RAD21*	0.190763	5.803668	12.67307	0.00037096	0.048132
*FUT8*	0.197327	5.011198	12.5474	0.000396759	0.049474
*CARD8*	0.223999	3.931231	16.5491	4.74E–05	0.014568
*S100PBP*	0.246188	4.671997	20.87633	4.90E–06	0.004005
*USP28*	0.249203	4.178656	20.00123	7.74E–06	0.005454
*BDH2*	0.253192	3.366615	14.47918	0.000141717	0.028533
*CASP4*	0.256899	4.924556	20.10625	7.33E–06	0.005253
*ZCCHC8*	0.260018	4.188864	20.62361	5.59E–06	0.004311
*B4GALT5*	0.262408	5.952676	14.25092	0.000159983	0.031285
*IRAK1BP1*	0.275405	2.588981	13.84753	0.000198258	0.034817
*IFI16*	0.278147	6.395011	14.08274	0.000174942	0.032973
*AMMECR1*	0.278167	4.031016	13.71351	0.000212917	0.035959
*SH2D4A*	0.295128	5.667351	13.02964	0.000306599	0.04321
*DPH5-DT*	0.301347	2.46994	16.22833	5.61E–05	0.016806
*TMEM116*	0.310949	2.121463	13.07266	0.000299638	0.04297
*DPYD*	0.325722	5.376649	13.1719	0.000284178	0.041779
*SRPX*	0.330869	5.435651	18.19592	1.99E–05	0.008993
*ARHGEF3*	0.366546	2.935045	16.02308	6.26E–05	0.017517
*SERAC1*	0.374418	5.547421	16.32552	5.33E–05	0.016149
*CDK14*	0.379537	5.705607	16.07847	6.08E–05	0.017248
*GLT8D2*	0.386548	4.708298	16.93926	3.86E–05	0.013037
*ESR1*	0.387471	1.004918	13.63949	0.000221478	0.036797
*FGF2*	0.388723	4.715732	17.35999	3.09E–05	0.011813
*PLSCR1*	0.393244	3.954516	19.12633	1.22E–05	0.006494
*RUNX1T1*	0.395472	3.164303	13.35076	0.000258318	0.039691
*IGFBP3*	0.398171	12.15029	16.00963	6.30E–05	0.017522
*FMNL2*	0.401245	4.579389	13.79527	0.000203849	0.035154
*ZEB1-AS1*	0.405514	1.250406	16.35924	5.24E–05	0.015982
*RHOBTB3*	0.421879	4.703756	19.29777	1.12E–05	0.006177
*PTPRE*	0.456301	2.718066	15.96502	6.45E–05	0.017804
*OR2A1-AS1*	0.460911	0.345043	14.55567	0.000136078	0.027729
*MISFA*	0.467977	–0.147	12.54327	0.000397636	0.049474
*SIM2*	0.487467	2.960039	16.63429	4.53E–05	0.014249
*CELF6*	0.557284	–0.13149	16.17211	5.78E–05	0.016806
*ABCA12*	0.579788	0.062118	14.17864	0.000166247	0.032202
*SEMA5A*	0.582161	6.489798	17.20552	3.35E–05	0.01235
*IRS2*	0.591112	4.466242	14.10983	0.00017244	0.032848
*LINC01277*	0.786045	–0.53122	14.16108	0.000167806	0.032351
*ZFPM2*	0.801454	0.340995	12.91085	0.000326684	0.045108
*TWIST2*	0.831999	4.611952	12.65027	0.000375512	0.048415
*KCNK15*	0.843858	2.00907	16.57159	4.68E–05	0.014505
*LYPLAL1-AS1*	0.870673	–0.54629	20.83813	5.00E–06	0.004005
*CTSK*	0.892649	4.827643	20.56693	5.76E–06	0.004358
*RARRES1*	0.89793	1.201195	33.30716	7.87E–09	4.59E–05
*CELSR1*	0.899855	3.295554	12.7837	0.000349652	0.04727
*EPHA3*	0.942838	2.566101	16.95059	3.84E–05	0.013037
*LINC00906*	0.952773	–1.22901	19.67965	9.16E–06	0.005941
*DENND2A*	0.959001	0.240724	13.41387	0.00024977	0.038963
*EIF4A2P3*	1.000457	–1.55854	14.77132	0.000121367	0.025479
*ABCA6*	1.018584	2.061196	13.22672	0.000275986	0.041179
*USP6*	1.140794	–0.65042	13.33082	0.000261079	0.039816
*C3*	1.160315	4.277584	13.64326	0.000221033	0.036797
*FOLR3*	1.242528	–1.2425	14.01678	0.000181187	0.033347
*LHX9*	1.30102	1.764174	32.56135	1.15E–08	5.24E–05
*ST3GAL6-AS1*	1.400417	–2.06166	12.94407	0.000320936	0.044663
*LINC02385*	1.512722	–1.89567	18.89287	1.38E–05	0.007153
*SLC15A5*	1.512989	–1.98155	17.51228	2.85E–05	0.011007
*CASP16P*	1.513554	–2.07019	13.84536	0.000198486	0.034817
*CNTNAP3B*	1.785434	2.465226	16.2065	5.68E–05	0.016806
*ZNF232*	3.397878	–2.27352	14.85184	0.000116294	0.024756

To conceptually validate the applicability of transcriptomic data analysis for predicting donors’ chronological age or the duration of cell cultivation, we prepared corresponding predictive regression models based on two approaches: LASSO regression and RFR. Genes were selected as predictors based on Pearson and Spearman correlation coefficients, in order to account for both linear and monotonic relationships between features and the target variables — chronological age and cultivation duration. As a result, the models based on LASSO regression and RFR have demonstrated close performance: R^2^=0.755; MAE=9.858 years, and R^2^=0.742, MAE=10.060 years, respectively (Table 5). The LASSO regression-based model has demonstrated the highest accuracy of the cell passage prediction on the test sample: R^2^=0.583; MAE=0.508 passages (Table 6).

**T a b l e 5:** Performance of predictive models based on LASSO regression and RFR for chronological age estimation

Indicators	RFR		LASSO	
	Pearson correlation coefficient	Spearman correlation coefficient	Pearson correlation coefficient	Spearman correlation coefficient
Number of genes (r>|0.5|)	26	135	26	135
MAE	10.516	10.060	9.858	12.731
R^2^	0.684	0.742	0.755	0.655

**T a b l e 6:** Performance of predictive models based on LASSO regression and RFR for the assessment of *in vitro* culture duration (passage number)

Indicators	RFR		LASSO	
	Pearson correlation coefficient	Spearman correlation coefficient	Pearson correlation coefficient	Spearman correlation coefficient
Number of genes (r>|0.5|)	26	186	26	186
MAE	0.490	0.440	0.508	0.682
R^2^	0.261	0.323	0.583	0.267

### Analysis of telomere length for cell senescence assessment

**Figure 2.**
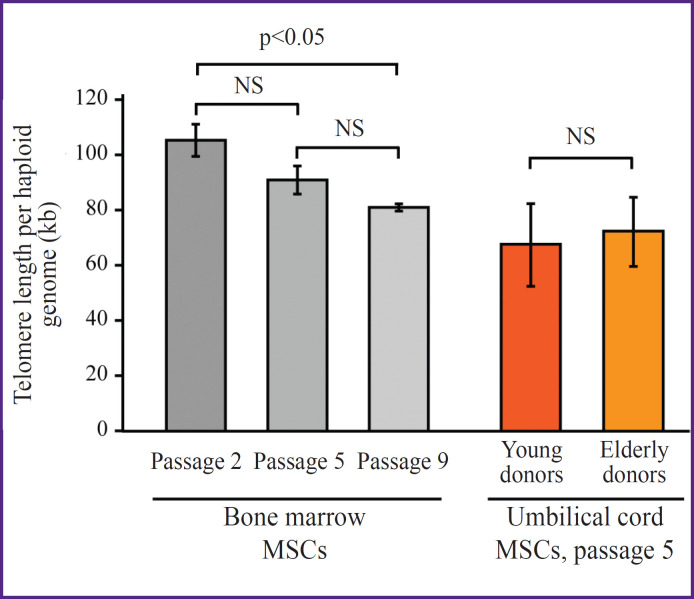
Comparison of the absolute telomere length normalized to the haploid genome in cultured MSCs Measurements were performed using quantitative PCR. The telomere length was compared between the umbilical MSCs at different passages and between the donor bone marrow MSCs isolated from the young (20– 25 years, n=3) and elderly (>60 years, n=3) donors. NS — p≥0.05, Student’s t-test

One of the traditional markers of assessing cellular aging is the analysis of telomere length. For this analysis we used the real-time PCR method. Samples of umbilical cord MSCs of different passages and BM-MSC samples from the donors aged 20–25 years (n=2) and older than 65 years (n=2) were analyzed on the sixth cultural passage. This method allowed to detect dynamics of the telomere shortening during MSCs cultivation. Statistically significant differences were observed after seven passages (Figure 2). At the same time, when samples from the donors of various ages were compared, no reliable differences were found.

### Analysis of nuclear morphology as a marker of cell senescence

**Figure 3.**
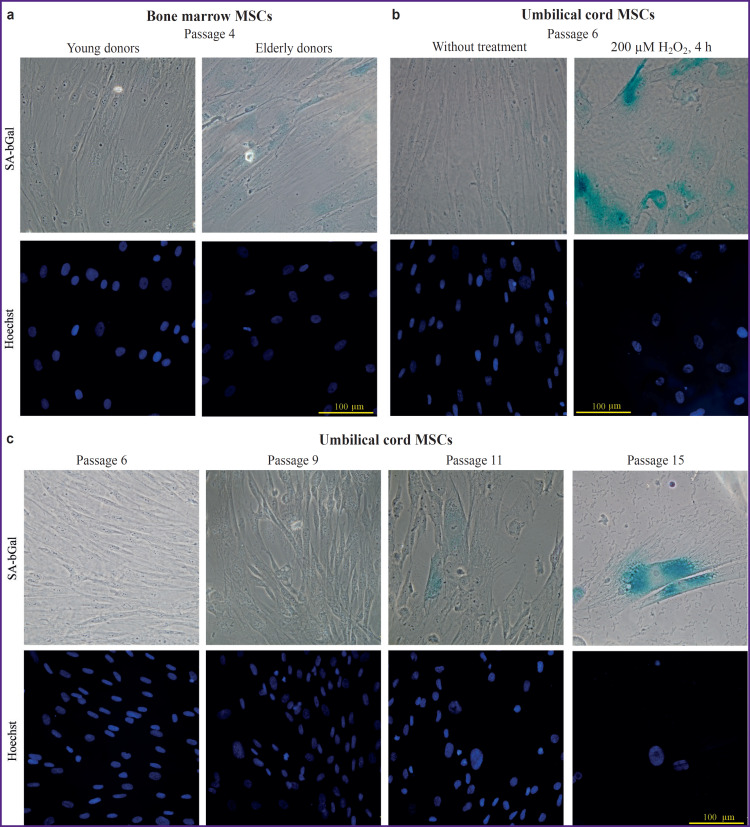
Investigation of senescence associated β-galactosidase (SA-bGal) activity in the cultivated MSCs Cells were fixed and stained to determine SA-bGal, nuclear DNA was stained with the Hoechst dye; (a) comparison of bone marrow MSCs from the young (18–25 years) and elderly (>65 years) donor at cultivation passage 4; (b) comparison of umbilical cord MSCs at passage 6 under standard cultivation conditions and after a 4-hour treatment of 200 μM solution of hydrogen peroxide; (c) comparison of umbilical cord MSCs at cultivation passages 6, 9, 11, and 15 under standard cultivation conditions

To evaluate the dynamics of the nuclear morphology and the expression of individual protein markers during the cell aging, we have analyzed BM-MSC samples of three donors from the young (18–25 years) and older age groups (over 65 years); umbilical cord MSCs exposed to continuous cultivation; and MSCs treated with hydrogen peroxide to induce senescence. In the cytochemical study of senescence- associated β-galactosidase activity (Figure 3), an increase in its activity was observed during replicative and stress-induced senescence (Figure 3, (b), (c)). When comparing MSCs obtained from the donors of different ages, the differences were not pronounced (Figure 3 (a)).

**Figure 4.**
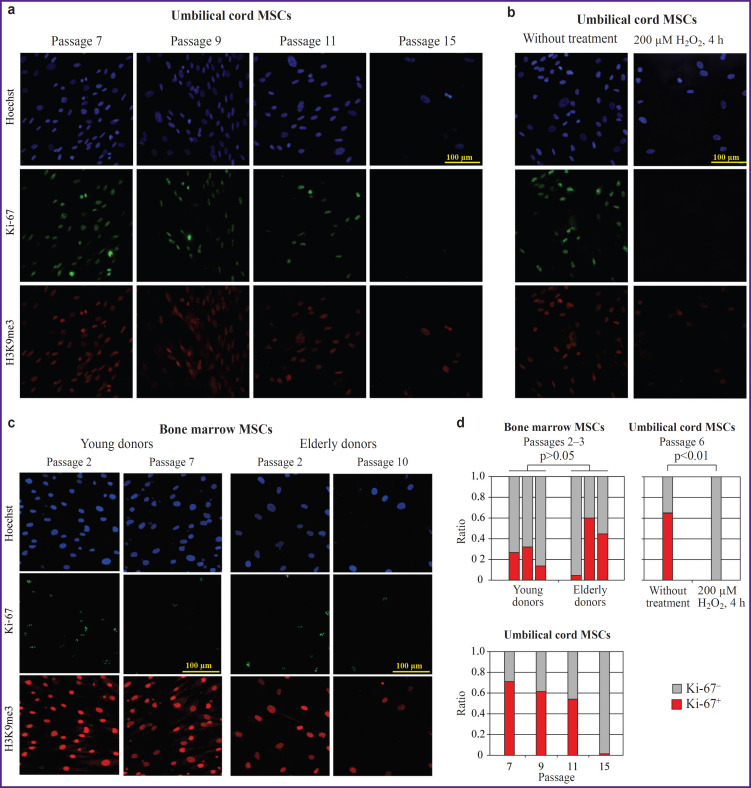
Investigations of the nuclear marker Ki-67 in the MSC cultures Formaldehyde-fixed MSC samples were stained with antibodies against the cell proliferation marker Ki-67 (a)–(c), the rate of Ki-67-positive nuclei on the samples was then determined (d). For visualization of nuclei, chromatin was stained with antibodies against histone modification H3K9me3, nuclear DNA was stained with Hoechst; (a) comparison of umbilical cord MSCs at passages 7, 9, 11, and 15; (b) comparison of umbilical cord MSCs at passage 6 under standard conditions and after 4-hour treatment of 200 μM solution of hydrogen peroxide; (c) comparison of staining the donor bone marrow MSCs at early and late cultivation passages; (d) the rate of Ki-67-positive cells on the stained MSC samples

Immunostaining analysis of the proliferation marker Ki-67 (Figure 4 (а)–(c)) allowed us to detect a relative decrease of the number of Ki-67-positive cells associated with cultivation duration (Figure 4 (a), (d)). Moreover, we did not find significant differences comparing cell preparations from the donors of various ages (see Figure 4 (c), (d)). However, at the level of nuclear morphology, the reduction of H3K9me3 signal intensity was noted, which agrees with heterochromatin erosion observed in aging, and enlargement of nuclei was observed at later passages and with increase of the donor age. Similar effects were also noted during continuous cultivation and stress-induced senescence of the umbilical cord MSCs (see Figure 4 (а)–(c)).

**Figure 5.**
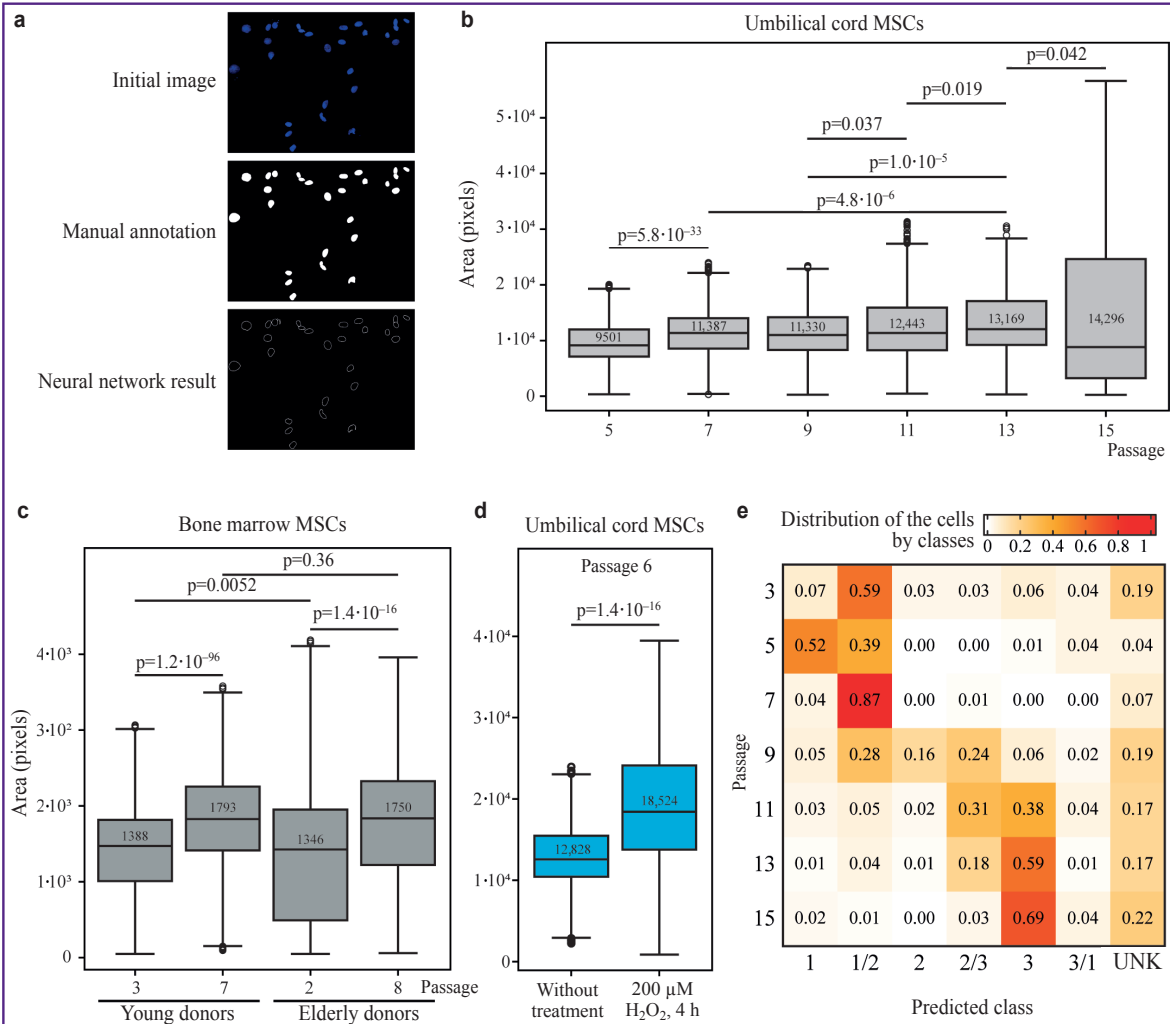
Investigation of the MSC samples using neural network algorithm for image segmentation and classification The image segmentation algorithm was trained based on the stained MSC samples, (a) upper image. For training the neural network, nuclear boundaries on the samples were manually annotated, (a) middle image. The trained algorithm successfully identified the nucleus boundaries, (a) lower image. The segmentation algorithm was subsequently used to estimate the sizes of MSC nuclei during passaging or under stress conditions (b, c, d), as well as for subsequent classification of cells into age classes (e). Figure (b) comparison of the nuclear areas in umbilical cord MSCs samples at passages 5, 7, 9, 11, 13, and 15; (c) comparison of nuclear areas in donor bone marrow MSCs samples at early and late cultivation passages; (d) comparison of the nuclear areas in umbilical cord MSC samples under standard cultivation conditions and after the 4-hour treatment of 200 μM solution of hydrogen peroxide; the numbers show mean area values; statistically significant differences were calculated using the Mann–Whitney test. Figure (e) a heat map of cell distribution across predicted classes in MSC samples from different passages. The neural network algorithm assigned cells to three classes: class 1 corresponded to early passages, class 2 to intermediate passages, class 3 to late passages. Cells that had an equal probability of belonging to two classes were assigned the value 1/2, 2/3, or 3/1. Cells for which a class could not be unambiguously determined were assigned the value “Unknown” (UNK)

It is interesting to note that an increasing amount of evidence is accumulating in favor of using changes in nuclear morphology as an independent marker of cellular senescence. From the technical point of view, this analysis looks robust, since it actually requires microscopic analysis coupled with fluorescent nuclear staining. Moreover, available approaches in the machine learning image recognition accelerate processing and collection of the required statistical data. For this reason, to systematically analyze changes in nuclear morphology during aging, we trained a segmentation neural network model that describes nuclear morphology parameters such as area, roundness, and ellipse parameters (see “Materials and Methods”). The model demonstrated high segmentation quality on the test set (Figure 5 (a)). As the primary metric for evaluating model performance, the Intersection over Union (IoU) measure was used. The IoU obtained for the model, equal to 0.88, indicates effective segmentation of cell nuclei and their boundaries even in the presence of noise and variations in the original images. For each nucleus, a set of parameters was calculated:

1. the center coordinates (X, Y) allow the nucleus to be matched with other cellular structures and used for spatial analysis; they are defined as the centroid derived from contour moments;
2. area — a marker of the overall size of the nucleus, an increase in area can be associated with cells transitioning to later passages;
3. roundness characterizes shape compactness, an increase in the value indicates that the nucleus is acquiring a more circular shape, which may be associated with later stages of cellular senescence;
4. the ellipse semi-axes and orientation angle allow assessment of the degree of elongation and the nucleus orientation;
5. the Hausdorff distance serves as a criterion of approximation quality, low values (<10) indicate that the nucleus shape conforms to an ellipse.

Using the developed model, data on the size and shape of MSCs nuclei (Figure 5 (b)–(d)) from the donors of various ages (n=9308), subjected to varying the duration of *in vitro* cultivation (n=5157), and before and after the induction of stress-mediated cell senescence (n=564) were collected. Cells from donors in the older age group showed a broader range of nuclear sizes at the early stages of culture, whereas with increasing passage the nuclear sizes of cells from donors of different ages converged (see Figure 5 (c)). During prolonged culture the nuclear size also increased significantly, and this increase occurred gradually (see Figure 5 (b)). Stress- induced senescence was associated with the most dramatic increase in nuclear size (see Figure 5 (d)).

At the next step, we evaluated the possibility of predicting the duration of cell cultivation, expressed as the ordinal number of the cultural passage, from nuclear morphology. The developed model classified the cells into three classes: early cultural passages (passages 3–5), intermediate (passages 7–9), and late (passages 11–15) according to the structure of the training dataset. As a result of the model’s operation, the analyzed cells were assigned a membership coefficient for one of the listed classes. The algorithm for calculating classmembership coefficients comprised several stages. At the first stage, a cell region was generated by merging the boundary mask with the class masks; then, based on the segmentation results, the cell contour was extracted and filled. The next stage involved counting overlaps with the class masks to determine the number of pixels that simultaneously lay within the cell region and the mask of the corresponding class. Subsequently, normalization by cell area was performed, whereby for each class the ratio of overlapping pixels to the total cell area was converted into a proportion ranging from 0 to 100%. These proportions were then adjusted so that their sum equaled 100%, and the corrected proportions were taken as the membership-coefficient values for each class. Membership to the primary classes (1, 2, 3) was defined by the highest coefficient value; a mixed class (1/2, 2/3, 3/1) was assigned when the difference between the coefficients of two classes did not exceed 10%; an undefined class was assigned to a cell when the coefficients for all classes were close to each other (all values below 40%). Evaluation of the algorithm on the test set demonstrated adequate prediction of the actual culture passages of the studied cells (Figure 5 (e)). Starting from passage 9, the representation of cells in different classes increased, which may be related to rising morphological heterogeneity of cells associated with aging. With increasing passage number in the test sample, the proportion of cells classified as late-passage cells steadily grew, most likely reflecting the dynamics of accumulation of senescent cells.

## Discussion

The key hallmark of cellular senescence is an irreversible arrest of the cell cycle mediated by the activation of cyclin-dependent kinase inhibitors p16^INK4a^ and p21^CIP1a^ [3]. In the studied models of MSC cellular senescence, the genes encoding the proteins p16^INK4a^, p21^CIP1a^ also displayed the expected dynamics. Moreover, substantial changes occurred in cell morphology and nuclear architecture. A significant role in the alteration of nuclear and chromatin structure is played by the senescence-associated decrease in the expression level of *LMNB1* gene [15, 34]. The reduction of the *LMNB1* gene expression level was observed in all examined models of cellular aging and it was most pronounced in the replicative senescence of MSCs. Specific changes in chromatin structure also include the formation of the so-called senescence- associated heterochromatin domains (SAHF and SAHD) together with decondensation of peri/centromeric heterochromatin regions (SADS) and global erosion of heterochromatin [16, 35–37]. These changes are involved in the regulation of both genes comprising the so-called senescence-associated proinflammatory phenotype [14, 35].

One of the factors determining the chromatin structure is a nuclear architectural protein, HMGB2, whose expression declines during cellular senescence and which, in particular, is considered as an early marker of cellular senescence [14, 38]. A decrease in the expression of the *HMGB2* gene was also detected with the increase of chronological age of MSC donors, in stress-induced and replicative senescence of MSCs. In the oncogene-induced senescence model HMGB2 has been shown to prevent propagation of heterochromatin in the genome regions containing genes forming the so-called senescence-associated proinflammatory phenotype, thereby helping to maintain their expression [14]. Although such an effect has not been confirmed in the replicative senescence, however, the development of a more permissive chromatin state during aging — caused by disruptions of the machinery that maintains facultative and constitutive heterochromatin, remodeling of the nuclear spatial topology, and activation of intracellular pro-inflammatory signaling pathways in response to DNA damage (cGAS-STING) — are key factors that determine the formation of the SASP [15, 37, 39].

The concept of senescence-associated secretory phenotype unites the complex of proinflammatory cytokines, growth factors, and metalloproteinases [40]. The main factors that constitute the SASP are TNFα, MCP-1, MCP-2, SERPINE1/PAI-1, GM-CSF, GROα, β, γ, IGFBP-7, interleukins IL-1α, IL-6, IL-7, IL-8, chemokine MIP1α, and matrix metaloproteinases MMP-1, MMP-10, and MMP-3 [41]. However, it is important to note that the composition of SASP changes significantly depending on the cause of cellular senescence and the cell type [18]. Interestingly, when comparing MSCs from donors of different ages, no significant dynamics in the expression of genes encoding individual interleukins was detected. At the same time, during culture and under stress- induced senescence, pro-inflammatory SASP factors such as IL-6, CXCL8, IL-1β became activated. This observation partially contradicts the previous reports of increased activity of these SASP factors in MSCs from older donors [42]. It should be emphasized, however, that in the cited study, a convincing difference in expression was demonstrated only for IL-6. Moreover, our analysis of published transcriptomes of BM-MSC samples (n=37) did not find reliable correlation between the expression of the investigated genes encoding individual SASP components and the age. All this together may reflect the heterogeneity and substantial contribution of donor-specific effects that complicate the analysis of age-related changes of gene expression. This is also supported by the performance of the regression model for predicting chronological age, which exhibited a relatively high value of the mean absolute error (R^2^=0.755; MAE=9.858 years).

It should be noted that previously described predictive models for estimating age from transcriptomic data have demonstrated comparable effectiveness [20, 43, 44]. More accurate similar algorithms generally achieve maximum performance on the narrow age cohorts. Moreover, in the process of model development, the authors excluded multiple available samples from the analysis, since their inclusion significantly worsened the model quality [45]. Thus, the evaluation of gene expression dynamics may be used to the greater extent to analyze cellular senescence *in vitro*. In this case, typically only limited number of cell lines are investigated under relatively standard cultivation conditions, which is likely to reduce the variability of gene expression profiles inherent to the primary donor cell cultures and samples. Similarly, according to the data obtained by us, the assessment of MSC telomere length, at least on the small sample sets, is also rather applicable for the evaluation of replicative senescence *in vitro*.

Cytological analysis techniques are widely used to study cellular senescence. Cellular senescence is accompanied by characteristic morphological changes such as flattening, enlargement of the cell and nuclear size, as well as the appearance of specific protein markers, like activation of senescence-associated β-galactosidase [3, 46]. In the present work, the activity of senescence-associated β-galactosidase demonstrated its applicability for the qualitative assessment of both replicative and stress-induced senescence. However, the use of this marker for the evaluation of the functional state of the cells requires standardization of several conditions. First, it is necessary to control the efficiency of the reagent lot used in the work, since the pH shift of these reagents may essentially distort the results. Taking into account the necessity to analyze the freshly prepared cell slides, it is not always feasible in serial experiments conducted in research laboratories. Besides, cell preparations must demonstrate similar cell density, since the elevated confluence can lead to distorted results [47]. Interpretation of the obtained results at the early stages of the cellular senescence may be difficult due to the absence of a fixed threshold value of the β-galactosidase activity, making it difficult to classify cells as positive or negative for this marker. Altogether, this limits the application of this marker for studying cell preparations obtained from donors of various ages.

As an alternative, approaches to assessing cellular aging based on the analysis of several protein markers associated with proliferation, apoptosis, and DNA damage may be considered [17]. At the same time, the applicability of these approaches is again limited by the selection of optimal markers. For example, expression of the protein Ki-67 widely used as a proliferation marker depends on the stage of the cell cycle, while the variant of γH2Ax histone, the marker of the DNA damage, is detected at the late stages of cellular senescence [17, 48].

With the development of the machine learning methods, there was proposed a concept, according to which the processive analysis of cell morphology may serve as an integral metric of cell aging [21, 49, 50]. To estimate the applicability to this approach, we have developed a segmentation neural network model for the automated assessment of the nuclear morphology parameters. The analysis of BM-MSCs from the donors of various ages at the early passages has shown that the range of nucleus sizes was wider in the cell sample of the donors from the older age group. The nucleus size gradually increased during MSC cultivation and the size of the BM-MSC nuclei from the donors of various ages did not demonstrate significant differences between the age groups. The most prominent change of the nuclear morphology was observed in stress-induced cell senescence. The developed model also allows for effective classification of the individual cell passage as a surrogate metric of the cell aging stage for umbilical cord MSC samples. In this connection, it may be supposed to be also employed for the estimation of the functional state of the donor BM-MSC samples if there is a sufficient amount of datasets for the training sample. Besides, the application of similar models to assess the effects directed to the reduction of cell senescence manifestations, such as rejuvenation by partial reprogramming is of great interest [51, 52].

## Conclusion

In the presented work, some aspects of phenotypic manifestations of various types of MSC senescence have been studied. At the level of individual gene expression, it has been shown that the change in the expression levels of *CDKN1A*, *LMNB1*, *HMGB2*, and *SERPINE1/PAI1* is observed in all investigated models of cellular senescence. At the same time, the analysis of transcriptomic data has demonstrated significant donor-dependent heterogeneity of gene expression profiles, which hampers creation of effective predictive models for the evaluation of chronological age and the duration of the *in vitro* cultivation. At the same time, an alternative predictive metric of cellular aging — at least in the case of replicative aging — can be changes in nuclear morphology, whose dynamic analysis using neural-network models allows us to estimate the duration of *in vitro* cultivation. Combining such approaches with other promising metrics, such as epigenetic clock algorithms, gives hope for developing functional algorithms to evaluate the phenomenon of cellular senescence.
